# Septin 9 isoform expression, localization and epigenetic changes during human and mouse breast cancer progression

**DOI:** 10.1186/bcr2924

**Published:** 2011-08-10

**Authors:** Diana Connolly, Zhixia Yang, Maria Castaldi, Nichelle Simmons, Maja H Oktay, Salvatore Coniglio, Melissa J Fazzari, Pascal Verdier-Pinard, Cristina Montagna

**Affiliations:** 1 Department of Genetics, Albert Einstein College of Medicine, Yeshiva University, 1301 Morris Park Avenue, Bronx, NY 10461, USA; 2 Department of Surgery, Jacobi Medical Center, 1400 Pelham Parkway South, Bronx, NY 10461, USA; 3 Department of Pathology, Jacobi Medical Center, 1400 Pelham Parkway South, Bronx, NY 10461, USA; 4 Department of Pathology, Albert Einstein College of Medicine, Yeshiva University, 111 East 210th Street, Bronx, NY 10467, USA; 5 Department of Anatomy and Structural Biology, Albert Einstein College of Medicine, Yeshiva University, 1301 Morris Park Avenue, Bronx, NY 10461, USA; 6 Department of Epidemiology & Population Health, Albert Einstein College of Medicine, Yeshiva University, 1301 Morris Park Avenue, Bronx, NY 10461, USA; 7 Centre de Recherche en Cancérologie de Marseille, Inserm U891, 27 bd Leï Roure, BP 30059, 13273 Marseille Cedex 09 France; 8 Institut Paoli-Calmettes, F-13009 Marseille, 27 bd Leï Roure, BP 30059, 13273 Marseille Cedex 09 France; 9 Aix-Marseille Université, F-13007 Marseille, 27 bd Leï Roure, BP 30059, 13273 Marseille Cedex 09 France

**Keywords:** Septin 9, breast cancer, oncogene, epigenetics, cytoskeleton

## Abstract

**Introduction:**

Altered expression of Septin 9 (*SEPT9*), a septin coding for multiple isoform variants, has been observed in several carcinomas, including colorectal, head and neck, ovarian and breast, compared to normal tissues. The mechanisms regulating its expression during tumor initiation and progression *in vivo *and the oncogenic function of its different isoforms remain elusive.

**Methods:**

Using an integrative approach, we investigated *SEPT9 *at the genetic, epigenetic, mRNA and protein levels in breast cancer. We analyzed a panel of breast cancer cell lines, human primary tumors and corresponding tumor-free areas, normal breast tissues from reduction mammoplasty patients, as well as primary mammary gland adenocarcinomas derived from the polyoma virus middle T antigen, or PyMT, mouse model. MCF7 clones expressing individual GFP-tagged SEPT9 isoforms were used to determine their respective intracellular distributions and effects on cell migration.

**Results:**

An overall increase in gene amplification and altered expression of *SEPT9 *were observed during breast tumorigenesis. We identified an intragenic alternative promoter at which methylation regulates *SEPT9_v3 *expression. Transfection of specific GFP-SEPT9 isoforms in MCF7 cells indicates that these isoforms exhibit differential localization and affect migration rates. Additionally, the loss of an uncharacterized SEPT9 nucleolar localization is observed during tumorigenesis.

**Conclusions:**

In this study, we found conserved *in vivo *changes of *SEPT9 *gene amplification and overexpression during human and mouse breast tumorigenesis. We show that DNA methylation is a prominent mechanism responsible for regulating differential *SEPT9 *isoform expression and that breast tumor samples exhibit distinctive SEPT9 intracellular localization. Together, these findings support the significance of SEPT9 as a promising tool in breast cancer detection and further emphasize the importance of analyzing and targeting SEPT9 isoform-specific expression and function.

## Introduction

Identification of biomarkers for early detection and new therapeutic targets of breast cancer helps to continuously reduce the morbidity of this frequent pathology in women. This entails resolving the physiological, cellular and molecular processes underlying the complexity of breast tumor development and associated tumor heterogeneity. One of the main sources of biomarkers and targeted chemotherapy is the cytoskeleton. Cytoskeleton-associated proteins are frequently misregulated during cancer initiation and progression and thus contribute to cancer cell proliferation, migration and invasion [1]. Septin guanosine triphosphatases that assemble into filaments represent such proteins. We previously identified Septin 9 (*SEPT9*), a cytoskeletal component [2], as a potential oncogene in breast tumorigenesis [3]. Analysis of a limited set of mouse mammary gland (MG) adenocarcinomas proposed *SEPT9 *as a novel oncogene because of its high level of genomic amplification in the form of double-minute chromosomes and jumping translocations. Such cytogenetic alterations act as a means of amplification for strong oncogenes, resulting in their overexpression and gain of function [4]. The role of *SEPT9 *as an oncogene is also supported by the findings that this locus is a hot spot for viral insertions that cause mouse T-cell lymphoma [5,6], and in human acute myeloid leukemia this gene is found to be a fusion partner of mixed lineage leukemia [7,8]. Since our original report, other research groups have shown that amplification and/or overexpression of *SEPT9 *occurs in ovarian carcinoma [9-11], head and neck cancer [12,13] and prostate cancer [14,15]. Additionally, SEPT9 downregulation results in the arrest of cell division in human mammary epithelial cells and human cancer cell lines [16,17].

The importance of *SEPT9 *as an early detection marker has recently been highlighted by the development of the diagnostic blood-based test for methylated *SEPT9 *DNA in colon cancer patients [18]. Nevertheless, the molecular mechanisms underlying the role of *SEPT9 *in tumor development are still largely unknown because of the expression of multiple isoforms [19], along with the fact that their modes of regulation have yet to be established. The oncogenic function of SEPT9 *in vivo *is still unclear, although SEPT9_v1 in particular has recently been shown to promote tumor growth by inducing angiogenesis via the stabilization of hypoxia-inducible factor-1 alpha (HIF-1α) in a heterotransplant model [14]. Other studies using breast nontumorigenic and cancer cell lines have indicated that different SEPT9 isoforms may have distinct cellular functions [20,21].

We performed a comprehensive and integrative analysis of *SEPT9 *gene amplification, expression at the mRNA and protein levels, and epigenetic profiling using a panel of human breast cell lines, tissues from breast cancer patients with premalignant lesions and primary adenocarcinomas, and normal breast tissue derived from reduction mammoplasty patients. We also used a mouse model for mammary tumorigenesis to examine progressive changes in *Sept9 *gene amplification and expression. Our data show that altered expression of *SEPT9 *isoforms is associated with stages of breast tumorigenesis and that changes in the isoform expression pattern do not occur solely by alternative splicing. DNA methylation of a putative alternative promoter region within *SEPT9 *demonstrates a regulatory mechanism that is responsible for the differing expression of at least one isoform in normal and cancer cells. In MCF7 clones expressing individual GFP-SEPT9 isoforms, we observed differential cytoplasmic versus nuclear SEPT9 localization corresponding to varying migration rates. Furthermore, in addition to its cytoplasmic localization, SEPT9 was also present in the nucleoli of human and mouse normal mammary epithelial cells, a localization that was lost in mammary carcinoma cells.

Collectively, our results demonstrate that *SEPT9 *is a marker for breast cancer that is worth investigating in large cohorts of patients and other cancer types. Such analysis will help to establish the significant genetic, epigenetic and proteomic signatures of *SEPT9 *in breast cancer and provide the basis for its use in early detection tests.

## Materials and methods

### Patient clinical data

Fluorescence *in situ *hybridization (FISH) and immunofluorescence analysis using tissue microarrays (TMAs) have been described previously [22]. Flash-frozen normal breast tissue, breast tumors and matching adjacent tumor-free areas used for real-time qRT-PCR and DNA methylation analysis were either collected at Jacobi Medical Hospital, kindly provided by Dr. Adrian L. Harris (Weatherall Institute of Molecular Medicine, University of Oxford, Oxford, UK), or obtained from the Cooperative Human Tissue Network (Additional file 1, Supplementary Table 1). The samples obtained for this study are part of an approved protocol for the collection of breast tissue samples (CCI protocol 2007-433, approved by the Albert Einstein College of Medicine Committee on Clinical Investigations) and were generated from leftover biopsies. Patient consent forms were obtained in compliance with the Declaration of Helsinki.

**Table 1:** Association between disease grade and copy number

Grade	Normal copy number	1 or 2 extra copies	3 or 4 extra copies	> 4 extra copies
BB (*n *= 7)	7	0	0	0
DCIS (*n *= 29)	16	8	4	1
Well diff. (*n *= 1)	1	0	0	0
Moderately diff. (*n *= 13)	2	5	5	1
Poorly diff. (*n *= 10)	2	6	1	1

p-value (Exact chi-square test for association) < 0.05; p-value (exact CMH test for non-zero correlation) = < 0.05

BB: benign breast; DCIS: ductal carcinoma *in situ*; diff.: differentiated.

### Mouse mammary gland adenocarcinomas

Mouse primary tumors were dissected from polyoma virus middle T antigen (PyMT) mice [23] (gift from Dr. Jeffrey W. Pollard, AECOM, Bronx, NY, USA) at the indicated time points. Tumor-bearing glands were isolated and cut in half. One portion was processed for Western blot analysis and genetic studies, and the other was embedded in paraffin. Four-weeks-old C57B6 female mice serving as normal controls were killed, and the fourth inguinal MGs were dissected. The use of murine models for this study was approved by the Institutional Animal Care and Use Committee at AECOM (protocol 20090502).

### Cell lines

The cell lines used for experiments are shown in Additional file 1, Supplementary Table 2. MCF10A cells were cultured as previously reported [24,25], hTERT-HME1 and 184A1 cells were cultured in mammary epithelium basal medium supplemented with the MEGM BulletKit (CC-3150; Lonza Group Ltd, Basel, Swtizerland) and all other cell lines were grown in DMEM supplemented with 10% fetal bovine serum. Because of the relatively high level of *SEPT9 *mRNA in the MCF10A cells, we analyzed the same cell line obtained from two different sources, Dr. Rachel Hazan (Department of Pathology, AECOM, Bronx, NY, USA) and the American Type Culture Collection (Manassas, VA, USA) (Additional file 1, Supplementary Table 2). HCT116 is a colorectal cancer cell line that was included in the analysis as a control for our DNA methylation data, since its DNA is highly methylated [26] and because of the prognostic role of DNA methylation of *SEPT9 *shown in colon cancer [27].

### Fluorescence *in situ *hybridization analysis of cell lines and tissue microarrays

Nine bacterial artificial chromosome (BAC) clones mapping to *SEPT9*, *HER2 *and CEP17 were obtained from the BACPAC Resources Center (Children's Hospital Oakland Research Institute, Oakland, CA, USA). Clones and their genetic mapping are shown in Additional file 2, Supplementary Figure S1A. BAC clones mapping to the mouse *Sept9 *gene were previously reported [3]. DNA isolation, labeling, hybridization and imaging were performed as described previously [22,28]. Slide imaging was carried out with a Zeiss Axiovert 200 inverted microscope (Carl Zeiss MicroImaging, Inc., Thornwood, NY, USA) using SpectrumOrange (SO ™)-specific (Abbott Molecular, Des Plaines, IL, USA), Cy5-specific and 4', 6-diamidino-2-phenylindole-specific filters (Chroma Technologies, Bellows Falls, VT, USA). Twenty-five cells were analyzed for each cell line, and locus-specific signals for SO (*SEPT9*) and Cy5 (*HER2*) were manually counted and plotted for further analysis. Unsupervised hierarchical clustering and heat map generation representing the signals for each cell were performed using R version 2.8 statistical software [29]. On TMAs, signals within each cell were counted for a minimum of 20 cells and scored as follows: two copies for each locus were classified as normal; cells with two copies of CEP17 and three to four copies of *SEPT9 *were classified as low copy number gain (+); cells with loss of CEP17 and at least two copies of *SEPT9 *and cells with two copies of CEP17 and five or more copies of *SEPT9 *were classified as moderately high copy number gain (++); cells with more than five copies of *SEPT9 *were scored as high copy number gain (+++).

Fisher's exact test of independence was used to test the null hypothesis of no association between grade and copy number, and the Cochran-Mantel-Haenszel test (based on Pearson's ρ) was generated to test the null hypothesis of no linear association, given the ordinal nature of both grade and copy number categories.

### Immunofluorescence on tissue sections and cell lines

Human TMAs (previously described), mouse mammary adenocarcinomas and age-matched normal tissues were baked at 60°C for 1 hour, deparaffinized in xylene (twice for 10 minutes each time) and rehydrated in a graded series of ethanol washes. Antigen retrieval was performed using Vector Antigen Unmasking Solution (H-3300; Vector Laboratories, Burlingame, CA, USA) for 20 minutes. Incubation with the following antibodies diluted in 3% goat serum/PBS was performed overnight at 37°C: anti-SEPT9 polyclonal antibody (gift from Dr. Koh-ichi Nagata, Institute for Developmental Research, Aichi, Japan [16]) detected with anti-rabbit Alexa Fluor 680 dye (Invitrogen, Carlsbad, CA, USA), monoclonal anti α-tubulin (DM1A clone; Sigma-Aldrich, St Louis, MO, USA) detected with anti-mouse Alexa Fluor 488 dye (Invitrogen); anti-nucleolin (gift of Dr. Thomas Meier, Department of Anatomy & Structural Biology, AECOM) detected with anti-mouse Alexa Fluor 488 dye (Invitrogen). All primary antibodies were used at a 1:1,000 dilution.

Stable MCF7 transfectants expressing GFP-tagged isoforms of SEPT9 were fixed in 3.7% formaldehyde for 10 minutes at room temperature. All images were acquired with an Olympus BX61 inverted microscope equipped with a UPlanSApo 63 × objective oil immersion lens (Olympus Imaging America, Inc., Melville, NY, USA), a mercury arc lamp for excitation, narrow band filters for all fluorescence emission, and a SensiCamQE CCD camera system (PCO AG, Kelheim, Germany) using a FISH view.

### Real-Time qRT-PCR

Total RNA was isolated from cancer cell lines, tissues and stably transfected GFP clones as reported previously [3]. cDNA was reverse-transcribed from 5 μg of total RNA using random primers and SuperScript II Reverse Transcriptase (Invitrogen). *SEPT9 *and GFP primers were designed with Primer3 software [30] (Additional file 1, Supplementary Table 3) along with glyceraldehyde 3-phosphate dehydrogenase (GAPDH) primers. *SEPT9_v1 *and *SEPT9_v3 *isoform primers were either as published [21] or designed by hand based on unique sequences at the 5' end. Real-Rime qRT-PCR was performed using Applied Biosystems Fast SYBR Green Master Mix and the StepOnePlus Real-Time PCR System (Life Technologies Corp., Carlsbad, CA, USA). Data normalization and analysis were performed as described previously [3]. *P *values for statistically significant differences in mRNA levels were calculated using a *t*-test.

### Western blot analysis of cell lines and tissues

Proteins (40 μg) were extracted from cell lines and tissues (Additional file 1, Supplementary Table 4) and separated on 7.5% acrylamide SDS-PAGE gels, then transferred to nitrocellulose membranes. Alternatively, NuPAGE Novex 4% to 12% Bis-Tris gradient gel and 3-(N-morpholino) propanesulfonic acid SDS running buffer (Invitrogen, Cergy-Pontoise, France) were used to achieve maximal resolution of SEPT9 bands. SEPT9 was detected by enhanced chemiluminescence using the anti-SEPT9 antibody (1:4,000 dilution) provided by Dr. Nagata [31], which recognizes human and mouse SEPT9_v1 through SEPT9_v4 isoforms and some uncharacterized high-molecular-weight isoforms; the antibody provided by Dr. Cossart (Institut Pasteur, Paris, France [32]) (1:1,000 dilution), which recognizes human SEPT9_v1 to SEPT9_v5 isoforms; or an antibody that recognizes only SEPT9_v3 [33] (provided by Dr. Tachibana, Osaka City University, Osaka, Japan) (Additional file 3, Supplementary Figure S2). Anti-α-tubulin DM1A antibody was used at a 1:2,000 dilution (Cell Signaling Technologies, Danvers, MA, USA), and anti-rabbit, anti-mouse and anti-rat secondary antibodies coupled to horseradish peroxidase were used at 1:2,000 dilutions (Jackson Immunoresearch Laboratories, Suffolk, UK). Quantification of bands was performed with the ImageJ software package (National Institutes of Health, Bethesda, MD, USA) and normalized to α-tubulin [34]. All positive bands between 37 and 75 kDa were counted as the SEPT9-related signal based on the expected molecular weight range defined by the five known isoforms (Additional file 1, Supplementary Table 5), and this signal was normalized to the α-tubulin signal at 50 kDa.

### Cloning of GFP-*SEPT9 *isoform fusions and generation of MCF7 stable transfectant clones

The *SEPT9_v1*, *SEPT9_v2*, *SEPT9_v3*, *SEPT9_v4 *and *SEPT9_v5 *isoforms [GenBank:AF189713, GenBank:AJ312319, GenBank:AF123052, GenBank:AJ312322 and GenBank:AJ312320] were cloned from normal breast cDNA (Clontech Laboratories, Inc., Mountain View, CA, USA) using an isoform-specific forward primer containing a *Xho*I cassette and a reverse primer common to all isoforms containing a *Hin*dIII cassette (Additional file 1, Supplementary Table 3). Two micrograms of isoform-specific plasmid were transfected into subconfluent MCF7 cells using FuGENE 6 Transfection Reagent (Roche Applied Science Indianapolis, IN, USA). Forty-eight hours after transfection cells were selected with 800 μg/mL G418 for 14 days. mRNA and protein levels were quantified for four clones per *SEPT9*-specific isoform (total of 20 clones) by real-time qRT-PCR and Western blot analysis (Additional file 4, Supplementary Figure S3).

### Cloning of *SEPT9_v3 *promoter and luciferase assay

**Figure 4:**
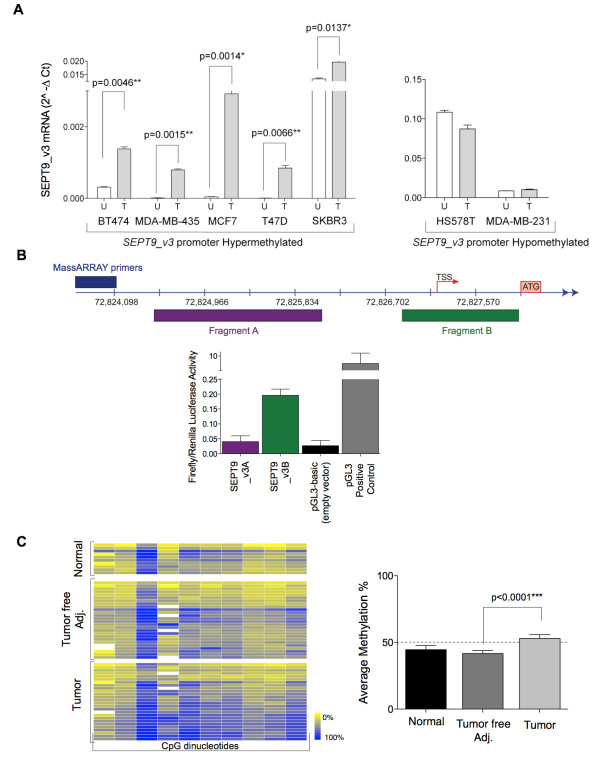
**Hypermethylation of the *SEPT9_v3 *promoter occurs in human breast primary carcinomas**. **(A) **Cell lines were treated with 5-aza-2'-deoxycytidine, and *SEPT9_v3 *mRNA was quantified. Representative cell lines with hypermethylation (left graph) and hypomethylation (right graph) of the *SEPT9_v3 *promoter region are displayed. U: untreated cells; T: treated cells. **(B) ***SEPT9_v3 *promoter region activity. Top: Location of tested luciferase fragments relative to the DMR. Bottom: Quantification of these fragments via luciferase reporter assay. **(C) **Supervised clustering of MassARRAY DNA methylation profiles in human primary adenocarcinomas, matching tumor-free areas and normal breast tissues. Right graph: Average levels of DNA methylation between tumors and tumor-free matching samples show a statistically significant difference.

The genomic region chr17:72,824,475-72,827,999 of the hg18 build of the human genome was selected based on mammalian conservation and the publicly available ENCODE Project integrated regulation tracks [35]. The fragments shown in Figure 4B were cloned into the pGL3-Basic Luciferase Reporter Vector (Promega, Madison, WI, USA) and transfected into MCF7 cells, where lysates were quantified with the Dual-Luciferase Reporter Assay System (Promega). Data from three technical and two biological replicates were plotted for a total of six data points for each fragment.

### Quantitative DNA methylation analysis using the MassARRAY EpiTYPER

Primers within the *SEPT9_v3 *promoter region were designed to include and target 10 different CpG sites using the MethPrimer program (Li Lab, University of California San Francisco, San Francisco, CA, USA) [36]. Matrix-assisted laser desorption/ionization time-of-flight mass spectrometry was performed using the MassARRAY EpiTYPER assay platform (SEQUENOM Inc., San Diego, CA, USA) on bisulfite-converted DNA as previously described [37]. For bisulfite conversion of DNA, we used the Zymo DNA purification kit (Zymo Research Corp., Irvine, CA, USA). MassARRAY primers (SEQUENOM Inc.) were designed to cover the genomic region HSA17 using the hg17 build of the human genome (chr17:72,823,233-72,825,460) [38] (Additional file 1, Supplementary Table 3). For 5-aza-2'-deoxycytidine treatment, cell lines were plated in 100-mm Petri dishes, allowed to attach overnight and treated with 2 μM 5*-*aza-2'-deoxycytidine for three days. Fresh 5-aza-2'-deoxycytidine stock was made every day and replaced together with media. Statistical analysis of the differences in methylation percentages between paired tumor and adjacent normal tissue samples at each CpG site was performed using nonparametric Wilcoxon signed-rank tests. *P *values were adjusted using the Holm-Bonferroni step-down method to correct for multiple testing [39]. The average methylation percentage across all CpG sites was compared in tumors versus tumor-free adjacent tissues as well as normal tissues using a paired *t*-test. Regression analysis to determine the coefficient of correlation between *SEPT9_v3 *mRNA and DNA methylation levels was performed using Microsoft Excel version 11.6.3 software (Microsoft Corp., Redmond, WA, USA).

### Migration assay and colony size assessment

Transwell inserts (8 μM; Millipore, Billerica, MA, USA) were coated with 25 μg/mL type I rat tail collagen (BD Biosciences Franklin Lakes, NJ, USA). Cells (5 × 10^4 ^in DMEM 0.3% BSA) were added in triplicate for each sample to the transwell inserts and allowed to migrate for 10 hours at 37°C. Nonmigratory cells were removed and filters were fixed in 3.7% formaldehyde/PBS for 15 minutes and stained with 0.2% crystal violet dye for 10 minutes. Migrated cells were counted and averaged from 10 fields of view per filter at 20 × magnification using an Axio Observer.A1 inverted microscope (Carl Zeiss MicroImaging, Inc.). Two independent biological replicate assays were analyzed. For cluster analysis, cells were seeded at 3.8 × 10^3^/cm^2 ^and allowed to grow for 5 days. The number of cells comprising clusters found within one-fourth of a 60-mm plate was counted for each cell line, again with the Axio Observer.A1 inverted microscope at 20 × magnification.

## Results

To determine how altered *SEPT9 *DNA copy number in human breast carcinogenesis might affect gene expression and protein translation, we performed a comprehensive analysis of DNA, mRNA and protein expression in a panel of 14 established and well-characterized human breast cancer cell lines. To potentially correlate *in vitro *with *in vivo *findings, 215 samples consisting of breast tumors, matched tumor-free tissues and normal breast epithelium were analyzed (Additional file 1, Supplementary Table 1).

### *SEPT9 *is amplified in breast cancer cell lines and human breast adenocarcinomas

**Figure 1:**
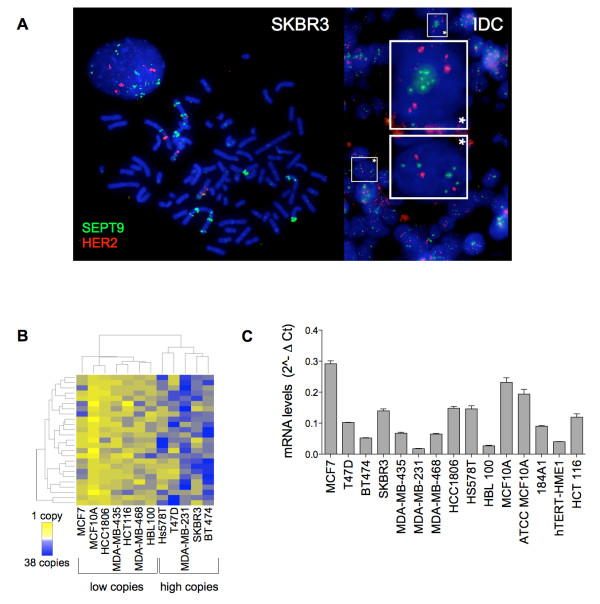
***SEPT9 *is amplified in human breast cancer**. **(A) **FISH labeling of *SEPT9 *gene (green) and *HER2 *gene (red) in SKBR3 breast cancer cells (left panel) and primary invasive ductal carcinoma (IDC) breast tissue (right panel). **(B) **Unsupervised hierarchical clustering of *SEPT9 *DNA copy number in human cell lines. Each block represents one cell, and 25 cells were counted for each cell line. **(C) **Total *SEPT9 *mRNA levels in the human cell lines (Additional file 1, Supplementary Table 2).

Using FISH to determine DNA copy number, a locus-specific probe for *SEPT9 *was cohybridized with a probe for *HER2*, a well-established oncogenetic biomarker that maps to the same chromosome arm as *SEPT9 *(Additional file 2, Supplementary Figure S1A). Gene amplification of both loci was observed in most cancer cell lines and human breast carcinoma samples (Figure 1A). The nontumorigenic epithelial cell line MCF10A exhibited the lowest copy number for both *SEPT9 *and *HER2 *oncogenes (2.6 and 2.28 average number of copies per cell, respectively) (Additional file 2, Supplementary Figure S1B). The nontumorigenic cell line HBL100, however, carries a higher average number of copies per cell for both genes (8.96 and 8.68), which is consistent with the knowledge that over time, cultured, immortalized cell lines derived from nontumorigenic breast tissue may acquire cytogenetic alterations similar to those of cancer cell lines [40]. Unsupervised clustering based on *SEPT9 *signals counted within each cell separated the 12 cell lines into low and high copy number clusters (Figure 1B). The low *SEPT9 *copy number group included the nontumorigenic HBL100 and MCF10A cell lines, while the high copy number group included the highly tumorigenic SKBR3 and MDA-MB-231 cell lines. Analysis of *SEPT9 *and *HER2 *copies for each cell line within a single cell revealed that the amplification of *SEPT9 *and *HER2 *represents two independent cytogenetic events (Additional file 2, Supplementary Figure S1C).

### *SEPT9 *is overexpressed at the mRNA and protein levels in breast cancer cell lines and human breast adenocarcinomas

We aimed to determine whether genomic amplification of *SEPT9 *results in increased mRNA levels in human breast cancer cell lines and whether this change translates into higher protein levels. Quantification of total *SEPT9 *mRNA levels (Figure 1C) showed that the cell lines expressed variable *SEPT9 *mRNA levels, but we found no direct correlation between mRNA levels and DNA copy number, suggesting that a more complex mode of regulation might occur at this genomic locus.

**Figure 2:**
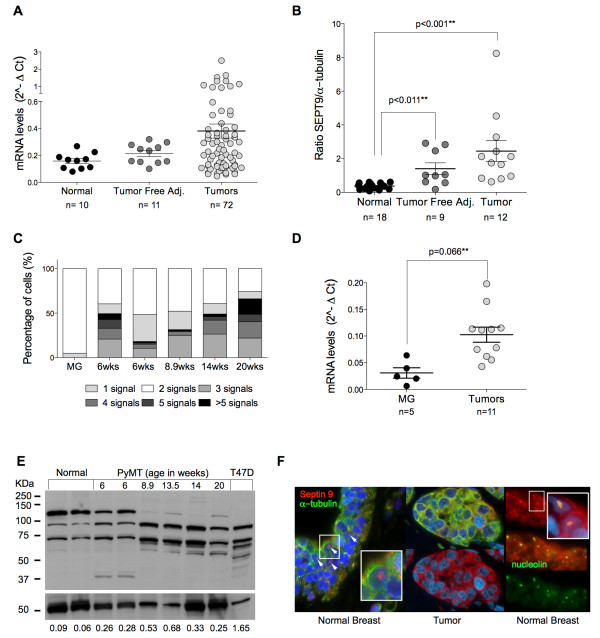
***SEPT9 *is amplified and overexpressed during breast tumorigenesis**. **(A) ***SEPT9 *mRNA levels quantified in primary breast tumors (light gray circles), tumor-free adjacent tissues (dark gray) and normal breast tissues from reduction mammoplasty (black circles). **(B) **Quantification of SEPT9 protein expression in breast primary tumors (light gray circles), tumor-free adjacent tissues (dark gray circles) and normal breast tissues from reduction mammoplasty (black circles). **(C) **Quantification of *Sept9 *gene copy number in normal mammary tissues and in primary mammary adenocarcinomas isolated from the PyMT mouse model at various stages of tumorigenesis (MG, normal mammary gland; 6 weeks premalignant hyperplasia, 8.9 weeks adenoma, and 14 and 20 weeks early and late carcinomas, respectively). Legend indicates copy number categories. **(D) ***Sept9 *mRNA levels in PyMT murine normal (black circles) and primary adenocarcinoma (light gray circles) mammary tissues. **(E) **Western blot depicting the pattern of Sept9 isoform (top panel) and α-tubulin (bottom panel) expression in normal mammary tissues and in primary mammary adenocarcinomas isolated from the PyMT mouse model at various stages of tumorigenesis. Values at the bottom indicate Sept9 expression levels normalized to α-tubulin. Only the bands between 37 and 75 kDa were used for quantification. T47D human breast cancer cell line in lane 9 was used as a reference to aid in isoform identification. **(F) **Left and center panels: immunohistofluorescence labeling of SEPT9 (red) and α-tubulin (green) in normal breast and adenocarcinoma tissues. White arrowheads indicate SEPT9 nuclear staining. Right panel: Colocalization of SEPT9 (red) and nucleolin (green) in normal breast tissues.

Because cytogenetic alterations of cell lines in two-dimensional culture systems may not accurately reflect what occurs *in vivo*, we examined the differences in *SEPT9 *expression in normal and tumor human breast tissues at the mRNA and protein levels. *SEPT9 *mRNA expression was quantified in 93 breast tissue samples consisting of normal tissue from individuals who had undergone reduction mammoplasty and tumors and their corresponding adjacent tumor-free breast tissue from cancer patients. There was a significant increase in *SEPT9 *in tumor tissues compared to both normal and tumor-free adjacent tissues (Figure 2A). Additionally, we analyzed SEPT9 protein content in breast tissues and tumors and found a significant increase in SEPT9 protein expression in tumors compared to normal tissues (Figure 2B and Additional file 5, Figure Supplementary S4B). Notably, we found higher expression in adjacent tumor-free tissues compared to normal tissues. This result suggests that either (1) SEPT9 protein expression might be influenced by the tumor microenvironment, thus affecting the adjacent tumor-free area of the breast, or (2) SEPT9 expression is altered early in the premalignant stage.

### *SEPT9 *gene amplification and expression changes during breast tumor progression

The observed differences in *SEPT9 *gene amplification and mRNA and protein levels in breast tumors compared to normal breast tissues prompted us to test whether these changes are associated with tumor grade. We analyzed *SEPT9 *copy number in a tumor set including benign tissues, ductal carcinoma *in situ *(DCIS) and breast tumors classified as poorly or moderately differentiated (grade II or III, respectively). For well-differentiated tumors (grade I), only one sample was available, and although it was included in the analysis, it was not used to draw conclusions. Only approximately one-half of the DCIS samples analyzed presented normal *SEPT9 *copy numbers, suggesting that genomic amplification of this oncogene occurs during early stages of tumorigenesis (Table 1). The maximum copy number detected in the primary tumors indicated that there are lower levels of gene amplification than those observed in breast cancer cell lines. The most significant finding of this analysis was that *SEPT9 *copy number increased in grades II and III, suggesting that high *SEPT9 *amplification correlates with poor clinical outcome.

To further examine whether *SEPT9 *gene amplification and increased *SEPT9 *mRNA and protein levels occur during tumor progression, we used the mammary carcinoma PyMT mouse model [41] to analyze stage-specific *Sept9 *expression. We detected *Sept9 *amplification in MGs with premalignant adenomas and observed that *Sept9 *gain (five signals per cell and higher) increases in advanced stage IV adenocarcinomas (Figure 2C). Similarly to human primary tumors, murine MG adenocarcinomas also expressed higher *Sept9 *mRNA (Figure 2D) and protein levels (Figure 2E) than normal MGs. At the protein level, a differential pattern of Sept9 isoform expression was reproducibly observed in normal tissue, hyperplastic tissue, adenoma and early and late carcinomas. There was an increase in Sept9 protein expression compared to normal MGs as early as the hyperplasia stage (six weeks). The most striking difference was a prominent decrease in the expression of a 120-kDa band with a concomitant increase in the expression of a 90-kDa band beyond hyperplasia (Figure 2E). Because these bands did not match any known isoform of Sept9, they were excluded from the quantification of Sept9 protein expression levels.

The observed quantitative changes in SEPT9 expression between normal tissue and breast tumors and during tumor progression impelled us to perform immunofluorescence on the same TMAs used for FISH to detect whether significant changes in SEPT9 intracellular localization occurred. All cells expressed characteristic SEPT9 cytoplasmic staining, but, surprisingly, normal breast tissues bore a strong nuclear signal in the luminal layer of the epithelium that was lost in tumor cells (Figure 2F and Additional file 1, Supplementary Table 6). We also analyzed MGs isolated from four-week-old virgin C57B6 mice and found similar intracellular localization (Additional file 1, Supplementary Table 6). Additionally, when we analyzed PyMT tissues at various stages of tumorigenesis, we found that approximately 40% of cells (all luminal) exhibited nuclear staining in hyperplasia, whereas only about 2% of cells carried a nucleolar signal in advanced tumor stages. On the basis of the morphology and size of the nuclear staining, it appears that SEPT9 localizes to the nucleolus of normal breast cells, which was confirmed by its colocalization with nucleolin (Figure 2F). Collectively, these results suggest that in normal breast tissue, SEPT9 maintains both cytoplasmic and nucleolar localization, whereas during tumorigenesis only cytoplasmic expression is retained.

### Hypermethylation of the *SEPT9_v3 *putative promoter is frequent in breast tumors and results in *SEPT9_v3 *silencing

**Figure 3:**
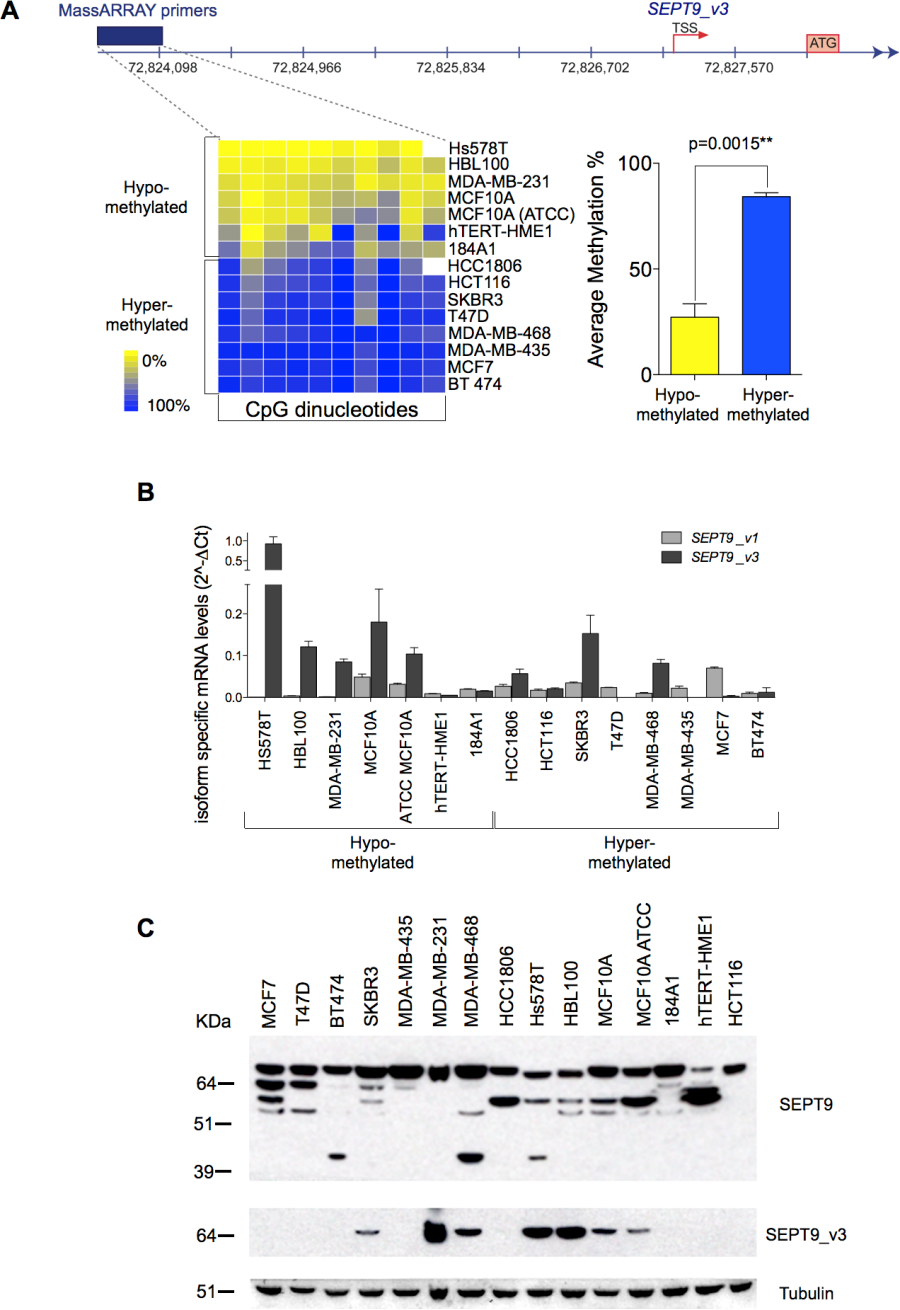
**Hypermethylation of an alternative promoter in breast tumors downregulates *SEPT9_v3***. **(A) **Quantification of DNA methylation at CpG sites upstream of the *SEPT9_v3 *transcription start site in cell lines targeted by MassARRAY. The unsupervised clustering distributes cell lines into two groups: hypomethylated and hypermethylated (left panel). Differences in the overall methylation percentages between these two groups are shown (right panel). White boxes represent CpG sites that were not analyzable. **(B) **Quantification of *SEPT9_v1 *and *SEPT9_v3 *isoform mRNA levels in human cell lines. **(C) **Expression of SEPT9 (top panel), SEPT9_v3 isoform (middle panel) and α-tubulin (bottom panel) detected by Western blot in the panel of breast cancer cell lines. Note that with the labeling of recombinant SEPT9_v1 and _v3 by an antibody recognizing both of these isoforms [16], the anti-SEPT9_v3 antibody recognizes the bottom band of the high-molecular-weight isoform doublet.

The lack of a direct correlation between *SEPT9 *copy number increases and mRNA levels prompted us to examine epigenetic modifications, specifically DNA methylation, at potential alternative transcription start sites (TSSs) in breast cancer. Alternative splicing of *SEPT9 *mRNA has previously been proposed as the primary mode of regulation of *SEPT9 *isoforms [19]. Recently, it has been suggested that DNA methylation of CpG sites near specific TSSs coding for individual isoform variants may be an alternative regulatory mechanism [18]. On the basis of our genomic and computational analyses of the mammalian *SEPT9 *gene, we found a highly conserved genomic region containing a CpG island that maps upstream to the *SEPT9_v3 *start codon, suggesting the presence of an alternative promoter (Figure 3A, top). We designed specific primers that targeted and quantified the methylation percentage at each CG dinucleotide within this region by using the highly sensitive MassARRAY technique on our panel of cell lines. Cell lines clustered into two groups, one group with the *SEPT9_v3 *TSS hypomethylated (average 18.4% methylation across all CpG sites) and one group with the *SEPT9_v3 *TSS hypermethylated (76.4%) (Figure 3A). To determine how DNA methylation alters *SEPT9_v3 *transcription, we quantified specific *SEPT9_v1 *and *SEPT9_v3 *mRNA levels in cell lines, using *SEPT9_v1 *for comparison since this specific isoform has been shown to increase in cancer (Figure 3B). As expected, *SEPT9_v1 *was expressed in most cell lines, whereas significant differences in mRNA levels were observed for the *SEPT9_v3 *isoform. A subset of cell lines (T47D, MDA-MB-435, MCF7 and BT474) expressed nearly undetectable levels of *SEPT9_v3*, whereas other cell lines (HS578T, HBL100, MDA-MB-231, MCF10A, SKBR3 and MDA-MB-468) expressed high levels. Interestingly, cell lines with hypermethylation at the *SEPT9_v3 *start site exhibited lower levels of *SEPT9_v3 *mRNA, whereas cell lines with hypomethylation at the *SEPT9_v3 *start site expressed higher *SEPT9_v3 *mRNA levels (*R*^2 ^= 0.678; significance deviation from zero: *P *= 0.0031) (Figures 3A and 3B). This finding also corresponded to repression of *SEPT9_v3 *expression at the protein level in cell lines that exhibited hypermethylation at the *SEPT9_v3 *start site (Figure 3C and Additional file 5, Supplementary Figure S4A).

To confirm the functional consequence of DNA methylation at the *SEPT9_v3 *start site on transcription, a subset of cell lines was treated with the DNA demethylating agent 5-aza-2'-deoxycytidine. As expected, treatment of cell lines displaying hypermethylation of the *SEPT9_v3 *start site resulted in increased *SEPT9_v3 *mRNA expression (Figure 4A, left graph), whereas it had no effect on cell lines exhibiting hypomethylation (Figure 4A, right graph). Taken together, these results strongly indicate that DNA methylation of an alternative promoter near the *SEPT9_v3 *TSS is a principal mechanism responsible for decreased *SEPT9_v3 *expression. The presence of an active alternative promoter (Figure 4B, green bar) was confirmed by performing a luciferase reporter assay in which the genomic region upstream of the *SEPT9_v3 *TSS was targeted (Figure 4B).

Next we analyzed the methylation status of the CpG sites proximal to the *SEPT9_ v3 *TSS in human breast tumor samples, their matching tumor-free adjacent areas and normal breast tissues from reduction mammoplasty patients (Figure 4C). MassARRAY analysis revealed an overall statistically significant difference in methylation levels between breast tumor tissues, the corresponding matching tumor-free area and normal breast tissues (52.9% versus 41.7% and 44.5%, respectively) (Figure 4C). Moreover, when DNA methylation was measured at each of the 10 individual CpG sites, we also found statistically significant differences in methylation levels between the tumors and the matching tumor-free tissue samples. These results suggest that *SEPT9_v3 *may frequently be silenced in breast tumors by methylation of its promoter region.

### SEPT9 isoforms differ in their respective cellular distribution and promigratory potential

**Figure 5:**
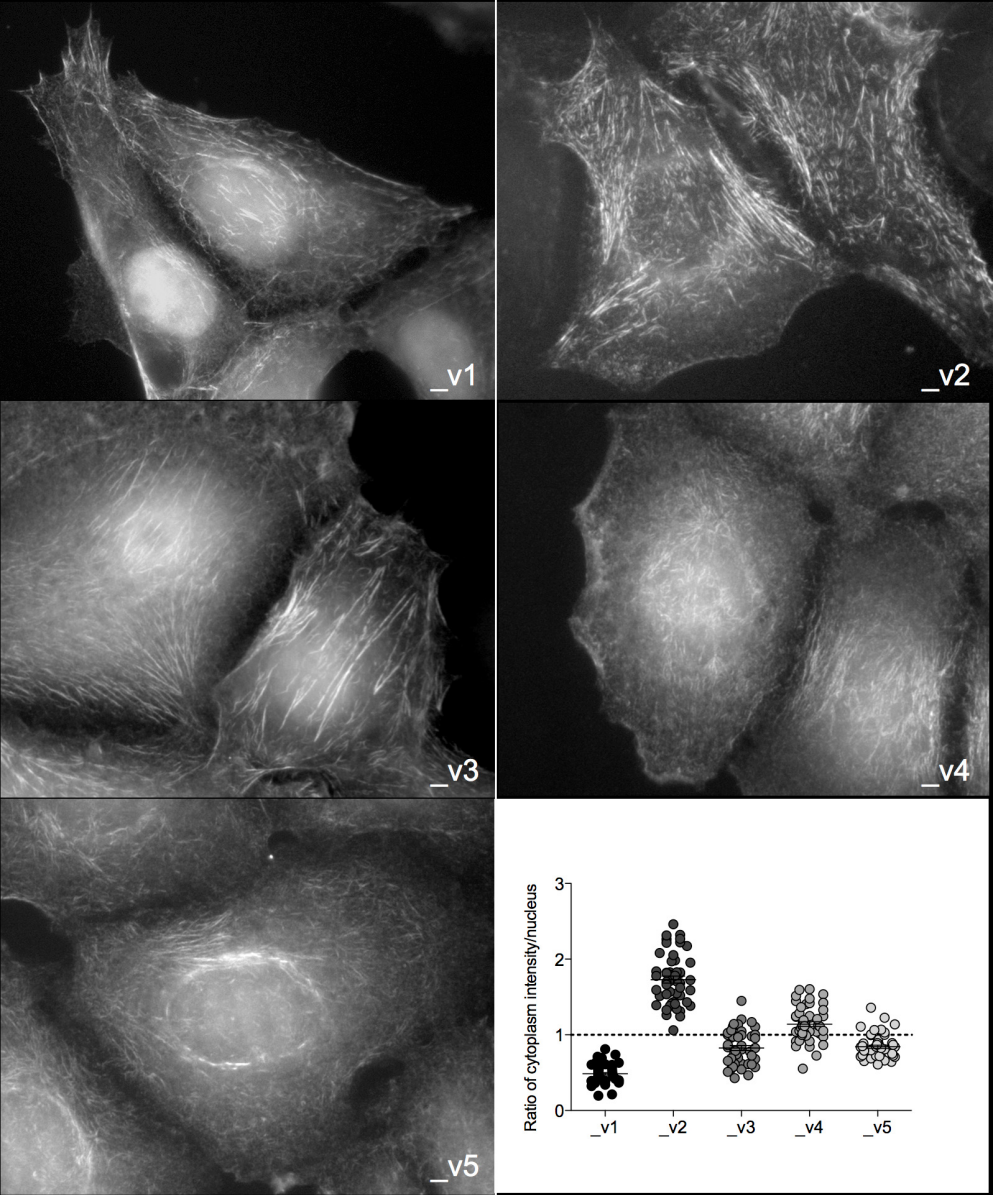
**SEPT9 isoforms localize in different cellular compartments**. Fluorescence microscopy of MCF7 clones expressing individual GFP-SEPT9 isoforms (gray scale). Expression of GFP-SEPT9 isoform constructs was quantified and plotted as a ratio of intensities measured outside the region occupied by the nucleus to measurements within the nucleus (bottom right panel). Average ratios located above the dotted line indicate cytoplasmic expression and those below the line indicate nuclear expression.

We sought to determine whether each SEPT9 isoform has similar intracellular distribution by generating stable MCF7 clones, each expressing one GFP-tagged SEPT9 isoform (Additional file 4, Supplementary Figure S3). All GFP-SEPT9 constructs were expressed in septin-like filamentous form, confirming functionality (Figure 5). Differences were observed in the organization of SEPT9 filaments between the nuclear region and the rest of the cytoplasm, thus the distribution of nuclear and cytoplasmic immunofluorescence was quantified for each of the isoform clones. SEPT9_v1 was mainly expressed within the nucleus, whereas SEPT9_v2 localization was sparse in that region. The other *SEPT9 *isoforms were relatively equally distributed between the nucleus and the rest of the cytoplasm.

**Figure 6:**
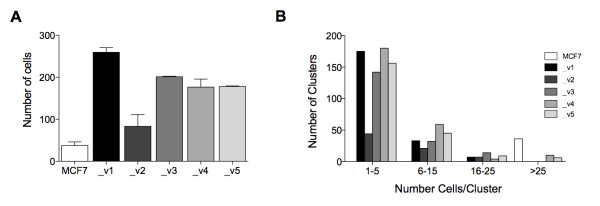
**SEPT9 isoforms alter migratory properties of MCF7**. **(A) **Transwell migration assay comparing MCF7 clones, each expressing a different GFP-SEPT9 isoform fusion construct. Plotting reflects the average number of migrated cells from two biological replicate assays conducted for each isoform. **(B) **Cell cluster size distribution for each MCF7 clone. The same gray scale is used in **(A) **and **(B) **to represent MCF7 and each isoform construct.

Because of the presumed specific oncogenic nature of *SEPT9_v1 *[21] and the differences in interphase cellular distribution of SEPT9 isoforms, we tested the promigratory potential of each SEPT9 isoform in our stable MCF7 clones (Figure 6A). GFP-SEPT9_v1, GFP-SEPT9_v3, GFP-SEPT9_v4 and GFP-SEPT9_v5 showed significant increases in the number of migratory cells, whereas GFP-SEPT9_v2, despite a similar mRNA expression level of total *SEPT9 *in cells (Additional file 4, Supplementary Figure S3D), was not as promigratory as the parental cell line. On the basis of this observation, we sought to determine whether increased migration is the result of a loss of cellular interaction, where single-cell growth is prevalent, as opposed to clustering, a common characteristic of epithelial cells. We counted the average number of cells present in a cluster for each GFP clone after five days of growth (Figure 6B). The data revealed that parental MCF7 tended to grow in clusters containing at least 25 cells. The least migratory isoform (GFP-SEPT9_v2) had the lowest number of cells per cluster in the 1-5 range compared to those that were most migratory (GFP-SEPT9_v1, GFP-SEPT9_v3, GFP-SEPT9_v4 and GFP-SEPT9_v5).

## Discussion

In this study, we performed a comprehensive analysis of the *SEPT9 *locus at the levels of DNA copy number, mRNA and protein expression in breast cell lines, normal human breast and breast tumor tissues, and normal mouse MGs and adenocarcinomas. We have shown that in humans, *SEPT9 *amplification varies at different stages of breast tumorigenesis and that in the PyMT mouse model it is dependent on the stepwise progression of mammary adenocarcinomas. Surprisingly, increases in *SEPT9 *gene copy number did not correlate with increased mRNA or protein expression in breast cell lines. However, in primary human and mouse tissues, more moderate *SEPT9 *gene amplification was observed, which correlated with elevated levels of *SEPT9 *mRNA and protein. Examination of SEPT9 localization by immunofluorescence to detect human normal breast tissues and breast tumors, as well as normal mouse and mammary adenocarcinoma sections, revealed that, aside from cytoplasmic staining, a specific nucleolar localization in normal tissue was present, primarily in the luminal layer of the ductal epithelium. Previous studies have shown that septins associate with the nuclear protein anillin during cytokinesis [42,43] and with karyopherins, a family of proteins involved in nuclear transport and septin SUMOylation [44]. Septins also interact with SOCS7 and accumulate in the nucleus to aid in DNA repair [45]. These studies, together with our results, suggest that septins may be involved in key nuclear processes contributing to normal cellular function and possibly to tumorigenesis.

Multiple modes of regulation are most likely responsible for controlling *SEPT9 *isoform expression, one of which, as we have shown, is epigenetic silencing via DNA methylation. A differentially methylated region (DMR) upstream from the *SEPT9_v3 *start site was identified where hypermethylation was shown to correlate with *SEPT9_v3 *silencing in cancer cell lines. Hypermethylation of this region was also observed in breast carcinomas. A luciferase reporter assay confirmed the presence of an active alternative promoter downstream from the DMR. Further investigation of this region will help to identify the transcription factors involved in the expression of *SEPT9_v3 *and how it is regulated during breast tumorigenesis. Our results suggest that expression levels of high-molecular-weight SEPT9 isoforms might be regulated by methylation at alternative promoter regions, and the current use of the methylation status of the *SEPT9_v2 *putative alternative promoter region as a marker for the early detection of colon cancer highly supports this hypothesis [18,46]. The role of methylation of CpG islands found in intragenic alternative promoters has been highlighted in a recent genome-wide study of the human brain, where this mode of regulation has been shown to be both tissue- and cell type-specific and is conserved in mouse [47]. Additional predicted alternative promoter regions must be studied to verify whether *SEPT9 *isoforms are regulated solely by DNA methylation or by alternative mechanisms as suggested previously [48]. We have found that estrogen receptor (ER)-positive tumors express significantly higher levels of *SEPT9 *mRNA than ER-negative tumors (data not shown), suggesting that alternative promoters found within the *SEPT9 *gene might be differentially regulated in breast cancer, potentially through epigenetic modifications or hormonal regulation.

When we compared normal mouse mammary tissue to mammary adenocarcinomas in PyMT mice, we observed increased expression of high-molecular-weight Sept9 isoforms (that is, isoforms SEPT9_v1, SEPT9_v2 and SEPT9_v3) during tumorigenesis. This finding is comparable to reports that suggest that SEPT9_v1 is one of the isoforms with oncogenic potential, as proven by different mechanisms. It stabilizes HIF-1α and increases during angiogenesis [14], and it stabilizes c-Jun N-terminal kinase and promotes proliferation [48]. Further identification and detailed mapping of posttranslational modifications by mass spectrometry are required, and they may identify high-molecular-weight isoforms such as those observed in mouse tissues (90 and 120 kDa). We did not observe a protein band matching the molecular weight of Sept9_v4 in the PyMT tissue. This was to be expected, because in the mouse, a valine residue replaces the first methionine of human SEPT9_v4. However, the fact that there was no Sept9_v4 expression in the PyMT mouse model suggests that SEPT9_v4 is not a key isoform in breast tumor development.

If different SEPT9 isoforms maintain specific cellular functions, changes in their respective expression levels could result in a gain of oncogenic properties. To test this hypothesis, we generated MCF7 clones expressing each of five isoforms individually. We observed that GFP-SEPT9_v1 was primarily localized within the nucleus, whereas GFP-SEPT9_v2 displayed mainly cytoplasmic localization, with the localization of GFP-SEPT9_v3, GFP-SEPT9_v4, and GFP-SEPT9_v5 being somewhat evenly distributed between both cellular compartments. *SEPT9 *was initially found to be overexpressed in human ovarian tumors with an observed increase in *SEPT9_v1 *and SEPT9_v4* isoform expression [10]. Our data, together with the findings of Petty's group [49], suggest that SEPT9_v1 is a key isoform in breast tumorigenesis that promotes cell migration. We observed that SEPT9_v2 influenced MCF7 cell migration the least and speculate that a regulatory mechanism such as methylation of its promoter prevents its expression, and therefore its function, in this cell line. The role of SEPT9 in the migration of normal and cancer cells is presumably connected to its association with microtubules and the actin cytoskeleton [16,31,50]. Intercellular junctions that interact with the cytoskeleton appear to be disrupted upon the overexpression of isoforms, causing an increase in the rate of migration, as shown by our results. A recent proteomic analysis of pseudopods isolated from metastatic cancer cell lines revealed that SEPT9 is enriched in these structures and that its downregulation induces a mesenchymal-epithelial transition [51], altering migration rates. SEPT9_v1 was found to exhibit nuclear localization and be the most promigratory isoform, whereas SEPT9_v2 was localized to the cytoplasm and affected migration rates the least. Further studies are necessary to determine whether cellular localization influences migration along with other cellular functions.

Collectively, our results strongly indicate that *SEPT9 *plays a role in tumorigenesis and that particular modes of regulation, such as epigenetic modifications, are involved in the differential expression of *SEPT9 *isoforms. An intriguing question raised by our study is the possible connection between the loss of SEPT9 nuclear localization and tumor development. What determines this change in intracellular localization, and does it contribute to aneuploidy, apoptosis and/or cancer cell invasion and metastasis? Furthermore, is there a balance in specific expression of *SEPT9 *isoforms and/or posttranslational modifications that drive localization and oncogenic potential? Our present and previous observations [3] indicate that *SEPT9 *operates as an oncogene in cancer cells, but it may also sustain a tumor-suppressing function when localized in normal epithelial nuclei.

## Conclusions

*SEPT9 *amplification occurs at the DNA level during human and mouse breast carcinogenesis and results in an overall increase in *SEPT9 *mRNA and protein levels. In this study, we have provided evidence that this process is associated with the alteration of *SEPT9 *isoform expression between normal and tumor cells in breast cancer. Moreover, we have shown that *SEPT9_v3 *isoform expression is regulated via DNA methylation at an alternative promoter, potentially driving changes in isoform expression during breast cancer initiation and progression. Our data support the hypothesis that *SEPT9 *isoforms maintain functional differences that could be selected for during tumor development and indicate that *SEPT9 *isoform profiling could lead to the development of additional biomarkers for breast cancer detection, prognosis and treatment. Therefore, this study provides a rational framework for further analysis of the expression of *SEPT9 *isoforms and their specific functions at the cellular and molecular levels in breast cancer development.

## Abbreviations

BAC: bacterial artificial chromosome; BSA: bovine serum albumin; DCIS: ductal carcinoma *in situ*; DMEM: Dulbecco's modified Eagle's medium; DMR: differentially methylated region; ER: estrogen receptor; FISH: fluorescence *in situ *hybridization; GFP: green fluorescent protein; HIF-1α: hypoxia-inducible factor-1 alpha; IDC: invasive ductal carcinoma; MG: mammary gland; PBS: phosphate-buffered saline; PyMT: polyoma virus middle T antigen; qRT-PCR: quantitative reverse transcriptase polymerase chain reaction; TMA: tissue microarray; TSS: transcription start site.

## Competing interests

The authors declare that they have no competing interests.

## Authors' contributions

DC carried out the methylation, real-time qRT-PCR and migration studies, as well as the immunofluorescence analysis, cell line and tissue sample collection and preparation, and edited and revised the manuscript. ZY performed the FISH and luciferase experiments and generated the GFP fusion clones. MC is the surgeon and NS and MHO are the pathologists who provided samples for analysis. SC supervised the migration assays. PVP performed the Western blot analysis and gave advice on manuscript preparation and revision. CM supervised the study and is responsible for the writing of the manuscript. All authors read and approved the final version of the manuscript.

## Supplementary Material

Additional file 1 **Supplementary Table 1**. List of breast tissue samples. Supplementary Table 2. List of cell lines used in the study. Supplementary Table 3. List of primer sequences. Supplementary Table 4. Western blot analysis protocol. Supplementary Table 5. Molecular weight of SEPT9 isoforms in human and mouse. Supplementary Table 6. Nuclear versus cytoplasmic localization. Click here for file

Additional file 2 **Supplementary Figure S1. *SEPT9 *is amplified in human breast cancer cell lines**. **(A) **Mapping of BAC clones selected for FISH analysis for *SEPT9*, *HER2 *and the subcentromeric region of chromosome 17. **(B) **Raw counts of the number of *SEPT9 *(green) and *HER2 *(red) signals detected by FISH in each of the 25 cells analyzed in our cell line panel. **(C) **The number of analyzed cells shown to exhibit increased *SEPT9 *copies (green), *HER2 *copies (red) or a balanced number of *SEPT9 *and *HER2 *copies (black) in human cell lines. Click here for file

Additional file 3 **Supplementary Figure S2. Sequence alignment of mouse and human SEPT9_v1 and mapping of antigens used to generate the antibody**. Click here for file

Additional file 4 **Supplementary Figure S3. GFP-*SEPT9 *isoform construct and expression**. **(A) **Vector map and ideogram depicting the cloning strategy used to generate GFP-*SEPT9 *fused isoforms. **(B) **Western blots of untransfected MCF7 and GFP_v1 through GFP_v5 fused clones. The membrane was probed with an anti-GFP antibody (top panel), with the anti-SEPT9 antibody provided by Dr Nagata (middle panel) and with α-tubulin (bottom panel). **(C) **Isoform-specific primers suitable for cloned cDNA were designed to uniquely amplify the *_v1 *through *_v5 *isoforms. These primers were used to confirm the specific overexpression of each isoform. **(D) **Real-Time qRT-PCR was performed to determine the level of *SEPT9 *overexpression of the clones (gray bars) compared to the parental MCF7 (white bar). Click here for file

Additional file 5 **Supplementary Figure S4. SEPT9 expression in human cell lines and breast tissues**. **(A) **SEPT9 expression in human breast cancer cell lines detected by Western blot analysis using Dr Nagata's antibody. **(B) **Western blot of matching human primary breast tissues and adjacent tumor-free area showing the expression of SEPT9 isoforms detected with Dr Cossart's antibody (top panel: Ponceau red, middle panel: SEPT9, bottom panel: α-tubulin). Click here for file
