# Supplementary material for: Septin 9 isoform expression, localization and epigenetic changes during human and mouse breast cancer progression
Source: Breast Cancer Res. 2011 Aug 10;13(4):R76. doi: 10.1186/bcr2924 (PMC3236340; doi:10.1186/bcr2924)
Supplement: Additional file 1 — Supplementary Table 1. List of breast tissue samples. Supplementary Table 2. List of cell lines used in the study. Supplementary Table 3. List of primer sequences. Supplementary Table 4. Western blot analysis protocol. Supplementary Table 5. Molecular weight of SEPT9 isoforms in human and mouse. Supplementary Table 6. Nuclear versus cytoplasmic localization. [file bcr2924-S1.DOC]

**Supplementary Table 1**

Summary of normal breast samples, primary breast tumors and tumor free matching adjacent tissues used for analysis.

| Source | Paraffin Blocks/TMAs | cDNAs | Flash frozen biopsies (cDNA-protein-DNA) |
| --- | --- | --- | --- |
| Montefiore Medical Center | 46 | 0 | 0 |
| Jacobi Medical Center | 0 | 0 | 33 (T)+33 (TFM) |
| University of Oxford | 0 | 83 | 0 |
| CHTN | 0 | 0 | 20 |
| Total samples/experimental procedure | 46 | 83 | 86 |
| Total number of samples analyzed | **215** | | |

T: tumor; TFM: tumor free matching adjacent area, CHTN: Cooperative Human Tissue Network

**Supplementary Table 2**

summary of cell lines used in the study and average number of sept9 and her2 dna copies/cell

| Cell line | Cancer type | ER | PR | HER2 | Tumor | Source | Invasive Boyden assay | Tumorigenicity | SEPT9  copies average/cell | HER2 copies average/cell |
| --- | --- | --- | --- | --- | --- | --- | --- | --- | --- | --- |
| MCF7 | Luminal | + | + | - | IDC | PE | - | Y | 8.36 | 5.68 |
| T47D | Luminal | + | + | - | IDC | PE | + | Y | 13 | 11.48 |
| BT474 | Luminal | + | + | + | IDC | PT | + | Y | 17.84 | 80.6 |
| SKBR3 | Luminal | - | - | + | AC | PE | - | Y | 13.76 | 36.88 |
| MDA-MB-435 | Basal | - | - | - | IDC | PE | nd | N | 6.48 | 10.6 |
| MDA-MB-231 | Basal | - | - | - | AC | PE | +++ | U | 18.04 | 17.4 |
| MDA-MB-468 | Basal | - | - | - | AC | PE | nd | Y | 8.76 | 7.64 |
| HCC1806 | Basal | - | - | - | DC | PT | nd | U | 5.04 | 6.2 |
| HS578T | Basal | - | - | - | C | PT | + | Y | 12.36 | 6.28 |
| HBL100 | Basal | - | - | - | non transformed | P Br | ++ | N | 8.96 | 8.68 |
| MCF10A | Basal | - | - | - | non transformed | P Br | - | N | 2.6 | 2.28 |
| hTERT-HME1 | U | - | U | - | non transformed | P Br | - | N | n.d | n.d |
| 184A1 | Basal | - | U | - | non transformed | P Br | - | N | n.d | n.d |
| HCT116 | Colorectal | U | U | U | Col C | PT | U | U | 7.72 | 6.8 |

ER: Estrogen Receptor; PR: Progesterone Receptor; IDC: Invasive Ductal Carcinoma; PE: Pleural Effusion; PT: Primary Tumor; AC: Adenocarcinoma; DC: ductal Carcinoma; C: Carcinoma, PBr: Primary Breast; Col C: Colon Carcinoma. U: unknown, n.d: not determined. Boyden assay: (-) non invasive; (+) low invasive; (++) highly invasive. Hs-578T and MDA-MB-435 were purchased from the NCI DCTD cell line repository; MDA-MB-468 was provided by A. Di Cristofano (AECOM); HCC 1806 and HBL100 were provided by L. Cerchietti (Weill Cornell Medical College); hTERT-HME1, HCT116 and SKBR3 were provided by Dr. T. Ried (NCI, NIH); 184A1 and MCF10A was provided by Dr. Rachel Hazan (AECOM), ATCC MCF10A were purchased from ATCC.

**Supplementary Table 3**

Primer sequences

| Primer name | Forward | Reverse |
| --- | --- | --- |
| sept9v1 | 5’-Ccg ctc gag cga tga aga agt ctt act cag gag g-3’ | 5’-Ccc aag ctt cta cat ctc tgg ggc ttc tgg ctc c-3’ |
| sept9v2 | 5’-Ccg ctc gag cgc cat gtc gga ccc cgc-3’ | 5’-Ccc aag ctt cta cat ctc tgg ggc ttc tgg ctc c-3’ |
| sept9v3 | 5’-Ccg ctc gag cgc cat gga gag gga ccg g-3’ | 5’-Ccc aag ctt cta cat ctc tgg ggc ttc tgg ctc c-3’ |
| sept9v4 | 5’-Ccg ctc gag cat gga gcc ccc tgc ct-3’ | 5’-Ccc aag ctt cta cat ctc tgg ggc ttc tgg ctc c-3’ |
| sept9v5 | 5’-Ccg ctc gag cat ggc cga cac ccc ca-3’ | 5’-Ccc aag ctt cta cat ctc tgg ggc ttc tgg ctc c-3’ |
| GAPDH | 5’-CCA CAT CGC TCA GAC ACC AT-3’ | 5’-cca ggc gcc caa tac g-3’ |
| GFP | 5’-CGA GCT GGA CGG CGA CGT AA-3’ | 5’-GCT TGC CGG TGG TGC AGA TG-3’ |
| GFPseptalliso | 5’-ACA TGG TCC TGC TGG AGT TC-3’ | 5’-ATG GAG TCA ATC CCC ACG TA-3’ |
| Seq1R |  | 5’-CTT GGG CAT CTG GAT CTC CA-3’ |
| Seq2F | 5’-ACT GCT GGC AGC CCA TCA TGA A-3’ |  |
| GFP1266F | 5’-ATG GTC CTG CTG GAG TTC GTG A-3’ |  |
| MSF1 | 5’-CCC GGT GGA CTT CGG CTA CG-3’ | 5’-GCT CTG CCC GAC CAC CAT GA-3’ |
| MSF2 | 5’-CAT CGC CAA GGC GGA CAC AC-3’ | 5’-CAC GTC GAT GCC GTT GGA CA-3’ |
| MSF | 5’-GCG GGA CCT TCT CAT CAG G-3’ | 5’-GAT GCT GCT GGT GAT GTC CT-3’ |
| Meth Primer 1 | 5’-AGG AAG AGA GTT GAG GGT TTT GTT TTT TTG GGA TA-3’ | 5’-CAG TAA TAC GAC TCA CTA TAG GGA GAA GGC TTT AAC CTA AAA TCC ATC TCC CCT CC-3’ |
| Petty_v1 | 5’-ATG AAG AAG TCT TAC TCA GGA GG-3’ | 5’-TGG GCC ACT GGA GTC ACC AAG C-3’ |
| v3 | 5’-ATG GAG AGG GAC CGG ATC TCA-3’ | 5’-CTC GGA GTA GGG GAG TCT GG-3’ |
| mSEPT9 | 5’-TGA AAG ATG CTC CCC GAT GT-3’ | 5’-CAT CTT CTT GCT TTG ACC TTC CTT-3’ |
| moGAPDH | 5’-CCC CCA ATG TGT CCG TCG TG-3’ | 5’-TGG GCC CTC AGA TGC CTG CT-3’ |

**Supplementary Table 4**

protocol for western analysis

| Steps |  | Buffer | Composition |
| --- | --- | --- | --- |
| 1 | Lysis | RIPA  (for cell lines 100ul of buffer for 100mm petri dish)  (for tissues 8ul of buffer for 100 mg of tissue) | 20 mM Tris-HCl pH 7.5, 150 mM NaCl, 1 mM Na_2_EDTA, 1 mM EGTA, 1% NP-40, 1% sodium deoxycholate, 2.5 mM sodium pyrophosphate, 1mM beta-glycerophosphate, 1 mM Na_3_VO_4_, 1 mg/ml leupeptin, protease inhibitors |
| 2 | Sonication | 10 sec | NA |

**Supplementary Table 5**

mw of human and mouse sept9 isoforms (Da)

| SEPT9 isoform | Human | Mouse |
| --- | --- | --- |
| _v1 | 65369 | 65575 |
| _v2 | 64649 | 64775 |
| _v3 | 63633 | 63773 |
| _v4 | 47469 | NA |
| _v5 | 38486 | 38599 |

**Supplementary Table 6**

nuclear localization in tissue sections (expressed as percentages)

| Tissue Sample Type | Nuclear and Cytoplasmic | Cytoplasmic Only |
| --- | --- | --- |
| Benign Breast (n=8) | 100 | 0 |
| DCIS (n=38) | 0.7 | 97.3 |
| IDC (n=39) | 0.6 | 97.4 |
| Mouse Normal Breast (n=6) | 100 | 0 |
| PyMT tumors (n=7) | 14 | 86 |
