# Supplementary figures and images for: Septin 9 isoform expression, localization and epigenetic changes during human and mouse breast cancer progression
Source: Breast Cancer Res. 2011 Aug 10;13(4):R76. doi: 10.1186/bcr2924 (PMC3236340; doi:10.1186/bcr2924)

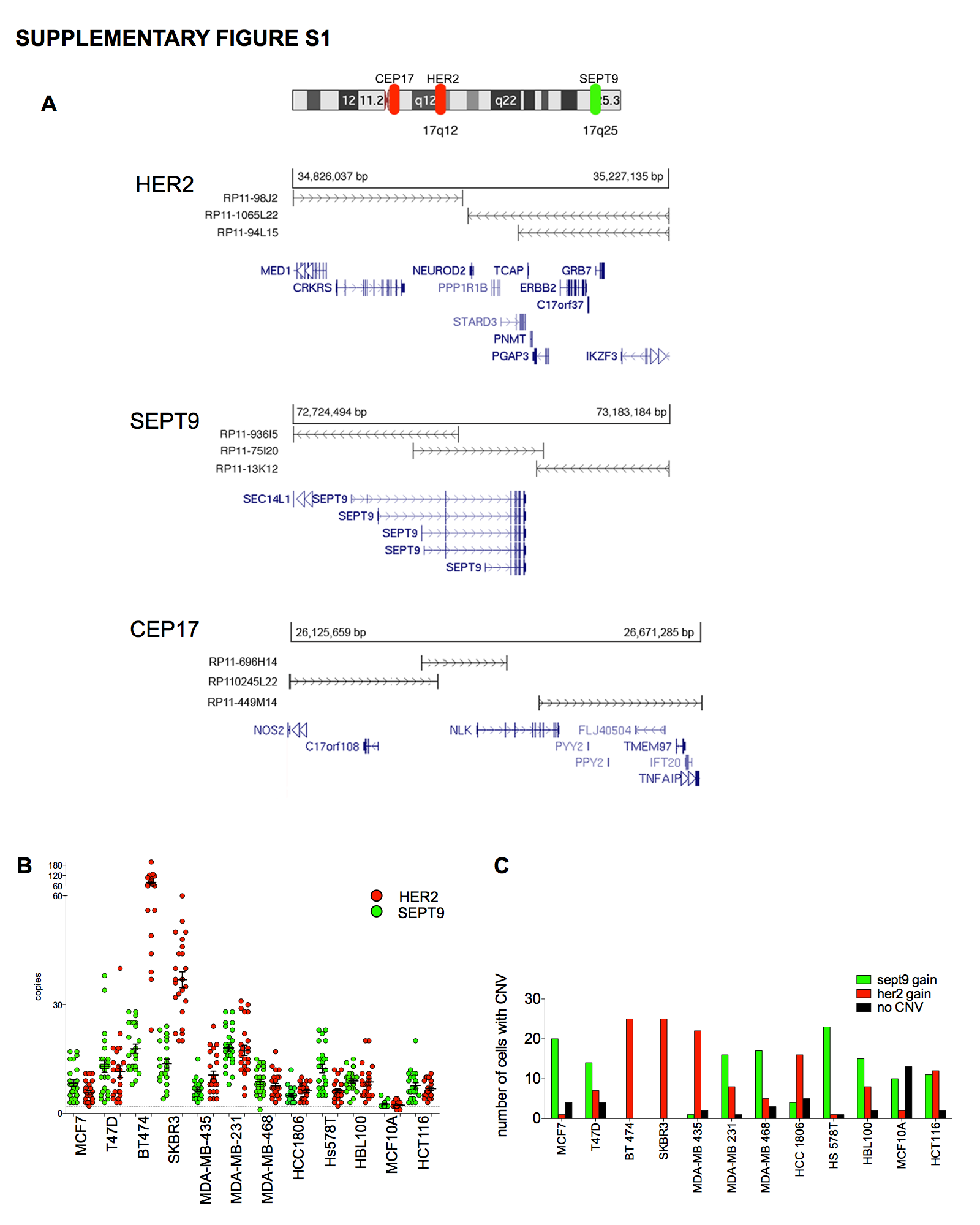

Supplement: Additional file 2 — Supplementary Figure S1. SEPT9 is amplified in human breast cancer cell lines. (A) Mapping of BAC clones selected for FISH analysis for SEPT9, HER2 and the subcentromeric region of chromosome 17. (B) Raw counts of the number of SEPT9 (green) and HER2 (red) signals detected by FISH in each of the 25 cells analyzed in our cell line panel. (C) The number of analyzed cells shown to exhibit increased SEPT9 copies (green), HER2 copies (red) or a balanced number of SEPT9 and HER2 copies (black) in human cell lines. [file bcr2924-S2.TIFF]

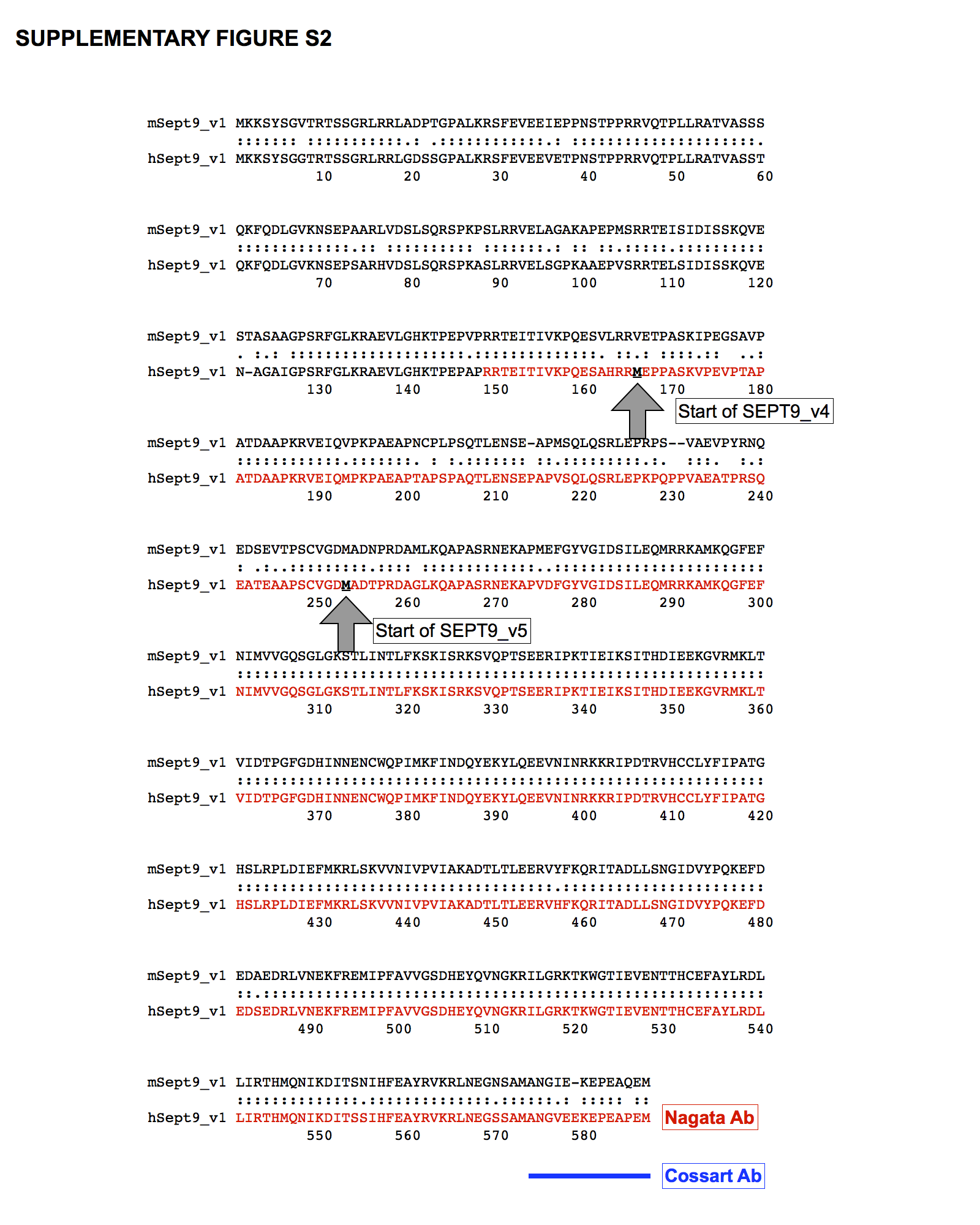

Supplement: Additional file 3 — Supplementary Figure S2. Sequence alignment of mouse and human SEPT9_v1 and mapping of antigens used to generate the antibody. [file bcr2924-S3.TIFF]

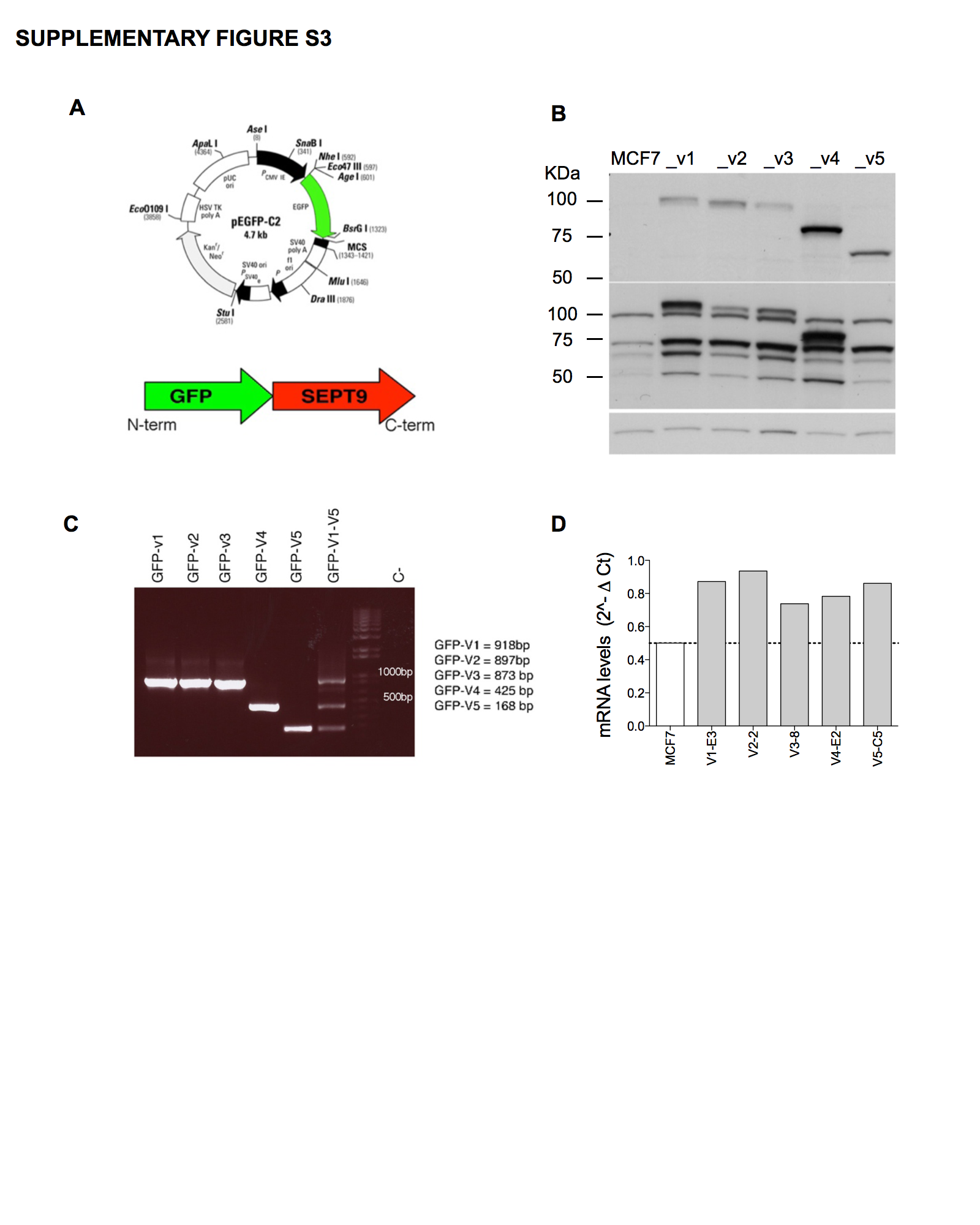

Supplement: Additional file 4 — Supplementary Figure S3. GFP-SEPT9 isoform construct and expression. (A) Vector map and ideogram depicting the cloning strategy used to generate GFP-SEPT9 fused isoforms. (B) Western blots of untransfected MCF7 and GFP_v1 through GFP_v5 fused clones. The membrane was probed with an anti-GFP antibody (top panel), with the anti-SEPT9 antibody provided by Dr Nagata (middle panel) and with α-tubulin (bottom panel). (C) Isoform-specific primers suitable for cloned cDNA were designed to uniquely amplify the _v1 through _v5 isoforms. These primers were used to confirm the specific overexpression of each isoform. (D) Real-Time qRT-PCR was performed to determine the level of SEPT9 overexpression of the clones (gray bars) compared to the parental MCF7 (white bar). [file bcr2924-S4.TIFF]

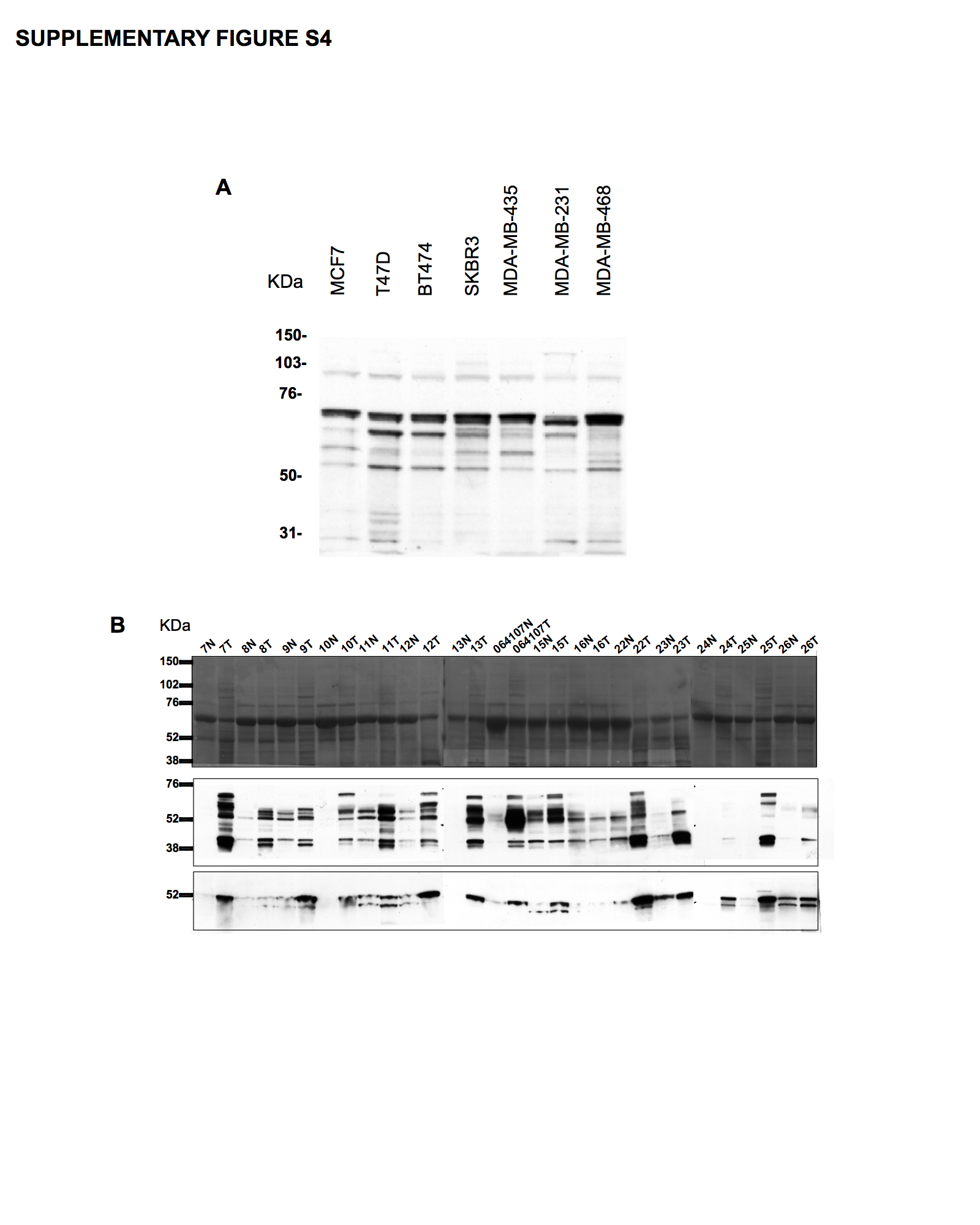

Supplement: Additional file 5 — Supplementary Figure S4. SEPT9 expression in human cell lines and breast tissues. (A) SEPT9 expression in human breast cancer cell lines detected by Western blot analysis using Dr Nagata's antibody. (B) Western blot of matching human primary breast tissues and adjacent tumor-free area showing the expression of SEPT9 isoforms detected with Dr Cossart's antibody (top panel: Ponceau red, middle panel: SEPT9, bottom panel: α-tubulin). [file bcr2924-S5.TIFF]
